# Immobilization of *Pleurotus eryngii* Laccase via a Protein–Inorganic Hybrid for Efficient Degradation of Bisphenol A as a Potent Xenobiotic

**DOI:** 10.3390/jox15040108

**Published:** 2025-07-03

**Authors:** Sanjay K. S. Patel, Rahul K. Gupta, Jung-Kul Lee

**Affiliations:** 1 Department of Chemical Engineering, Konkuk University, Seoul 05029, Republic of Korea; sanjaykspatel@hnbgu.ac.in (S.K.S.P.); guptarahul9m@gmail.com (R.K.G.); 2 Department of Biotechnology, Hemvati Nandan Bahuguna Garhwal University (A Central University), Srinagar 246174, India

**Keywords:** *Pleurotus eryngii* laccase, biocatalysts, bisphenol A, immobilization, inorganic–protein hybrids, xenobiotics

## Abstract

In the present investigation, an eco-friendly biocatalyst was developed using *Pleurotus eryngii* laccase (*Pe*Lac) through a copper (Cu)-based protein–inorganic hybrid system for the degradation of bisphenol A, a representative xenobiotic. After partial purification, the specific activity of crude *Pe*Lac was 92.6 U/mg of total protein. Immobilization of *Pe*Lac as Cu_3_(PO_4_)_2_–Lac (Cu–*Pe*Lac) nanoflowers (NFs) at 4 °C resulted in a relative activity 333% higher than that of the free enzyme. The Cu–*Pe*Lac NFs exhibited greater pH and temperature stability and enhanced catalytic activity compared to free laccase. This enhanced activity was validated through improved electrochemical properties. After immobilization, Cu–*Pe*Lac NFs retained up to 8.7-fold higher residual activity after storage at 4 °C for 30 days. Free and immobilized laccase degraded bisphenol A by 41.6% and 99.8%, respectively, after 2 h of incubation at 30 °C. After ten cycles, Cu–*Pe*Lac NFs retained 91.2% degradation efficiency. In the presence of potent laccase inhibitors, Cu–*Pe*Lac NFs exhibited a 47.3-fold improvement in bisphenol A degradation compared to free *Pe*Lac. Additionally, the synthesized Cu–*Pe*Lac NFs demonstrated lower acute toxicity against *Vibrio fischeri* than Cu nanoparticles. This study presents the first report of *Pe*Lac immobilization through an eco-friendly protein–inorganic hybrid system, with promising potential for degrading bisphenol A in the presence of inhibitors to support sustainable development.

## 1. Introduction

Laccases, multicopper oxidases belonging to the broader family of blue copper proteins, have garnered significant attention across various industrial sectors due to their ability to catalyze the oxidation of a wide range of substrates—particularly phenols and aromatic amines—by utilizing molecular oxygen as the electron acceptor and producing water as the sole byproduct [1,2]. This enzymatic capability makes laccases ideal candidates for applications such as bioremediation, pulp and paper processing, and the synthesis of novel biomaterials, offering a greener alternative to conventional chemical processes [3,4]. Laccases have been identified in diverse biological sources, including plants, bacteria, fungi, actinomycetes, and insects; however, fungal laccases are the most significant for biotechnological applications [5,6]. Despite their versatility, the practical use of free laccases on a large scale is often constrained by inherent limitations such as susceptibility to denaturation and non-reusability. Enzyme immobilization techniques have emerged as a promising approach to overcome these limitations, providing enhanced stability, reusability, and catalytic efficiency [7,8,9].

For immobilization, selecting an appropriate support matrix plays a critical role. Various immobilization strategies such as physical adsorption, covalent attachment, and encapsulation can be employed to immobilize laccases onto support matrices [10,11,12]. Physical adsorption, a relatively simple and cost-effective method, relies on non-specific interactions between the enzyme and the support surface [13]. Covalent attachment involves the formation of stable chemical bonds between the enzyme and the support, resulting in enhanced enzyme stability and reduced leaching [7]. Encapsulation traps the enzyme within a protective matrix, shielding it from harsh environmental conditions and preventing leakage [14,15]. Despite the advantages of laccase immobilization, several limitations and challenges warrant careful consideration. One of the primary challenges lies in maintaining the enzyme’s activity and substrate accessibility following immobilization [16,17]. The choice of support material can significantly influence the catalytic properties of the immobilized system. The immobilization process may induce conformational changes in the enzyme structure, thereby reducing activity or altering substrate specificity [18,19]. Additionally, mass transfer limitations—caused by restricted diffusion of substrates and products within the support matrix—can impede the overall catalytic efficiency of the immobilized enzyme [14,17]. Furthermore, the cost-effectiveness and scalability of the immobilization process are crucial factors to consider for industrial applications. The support material should be relatively inexpensive and environmentally benign, thereby minimizing the economic impact of the process [7,16]. The immobilization method must also be suitable for large-scale production, ensuring a sufficient supply of immobilized enzyme for practical use. The reusability of the immobilized enzyme is another critical factor that determines the overall economic viability of the process [20,21]. Although nanoparticles (NPs) offer several benefits for enzyme immobilization, their high toxicity remains a major concern for environmental sustainability [7,13]. Recently, integrating inorganic materials with proteins to create hybrid nanoflowers (NFs) has emerged as a promising approach with significant potential in biocatalysis and environmental applications [22,23]. Ge et al. reported the formation of protein–inorganic hybrids using copper (Cu) and various enzymes, including α-lactalbumin, laccase, carbonic anhydrase, and lipase [24]. In this mechanism, NFs are synthesized by incubating Cu or other metals with an enzyme in phosphate buffer via three steps: (i) nucleation and formation of primary crystals, (ii) crystal growth, and (iii) assembly into NFs. The type of metal such as Cu, cobalt (Co), nickel (Ni), zinc (Zn), and barium (Ba), as well as the incubation conditions (temperature and duration), significantly influence laccase properties after immobilization through protein–inorganic hybrids as NFs [8,20,21,23,25].

Bisphenol A, a ubiquitous environmental contaminant, has garnered significant attention due to its potential endocrine-disrupting effects and widespread presence in various consumer products, including polycarbonate plastics, epoxy resins, and thermal paper [26,27]. Its extensive use in the packaging industry, particularly in the lining of canned food products, has raised concerns regarding its potential impact on human health, thereby necessitating comprehensive investigation [28,29]. These concerns stem from the compound’s ability to mimic estrogen, potentially disrupting the endocrine system and leading to adverse health outcomes. It has been demonstrated that exposure to bisphenol A can induce estrogenic effects, which may result in reduced fertility, developmental abnormalities, and an increased risk of cancer. Consequently, there is growing interest in developing effective strategies for the degradation and removal of bisphenol A from the environment [30,31,32]. Laccase has diverse sources, including plants, bacteria, and fungi, with redox potential in the range of ~400 to 800 mV [33]. Therefore, the relatively higher redox potential of fungal laccases over plant and bacterial laccases, with their diverse applications, can be useful for immobilization studies. A few reports are available on the purification with a partial characterization of laccases from *Pleurotus eryngii* (*Pe*Lac) [34,35,36]. Still, protein–inorganic hybrid system-based immobilization of *Pe*Lac has not been reported.

In the present study, partially purified laccase from *Pe*Lac was used to synthesize Cu-based protein–inorganic hybrids as Cu–*Pe*Lac NFs through encapsulation, with the aim of enhancing the stability of the free enzyme and facilitating its application in the degradation of bisphenol A, a potent xenobiotic. Upon encapsulation as NFs, *Pe*Lac exhibited significant improvements in pH and temperature activity profiles, kinetic properties, and overall stability, while retaining high reusability. Furthermore, Cu–*P*eLac NFs were effectively applied for bisphenol A degradation under recycling conditions, even in the presence of potent inhibitors.

## 2. Materials and Methods

### 2.1. Materials, Chemicals, and Culture

Cu nanoparticles (Cu-NPs), CuSO_4_, 2,2′-azino-bis(3-ethylbenzothiazoline-6-sulfonate) (ABTS), dithiothreitol, and fluorescein isothiocyanate (FITC) were purchased from Sigma-Aldrich (St. Louis, MO, USA). Ultrapure water and phosphate-buffered saline (PBS) were obtained from Life Technologies (Carlsbad, CA, USA). A lyophilized culture of *Vibrio fischeri* was obtained from Modern Water (New Castle, DE, USA). All other analytical-grade reagents were purchased from commercial suppliers. *P. eryngii* KCCM 60470 was obtained from the Korean Culture Center of Microorganisms (Seoul, Republic of Korea).

### 2.2. Production and Partial Purification of PeLac

The culture of *P. eryngii* was grown on a potato dextrose agar plate. Fully grown mycelia (five disks of approximately 5 mm in diameter) were transferred to 100 mL of potato dextrose broth medium in a 0.5 L conical flask and incubated at 30 °C for five days under shaking conditions (175 rpm) to prepare the pre-culture. Thereafter, 10% (*v*/*v*) of the pre-culture and a laccase inducer (0.2 mM CuSO_4_) were added to 1.0 L of basal medium (pH 5.0), and enzyme activity was monitored over a ten-day incubation period [37]. For partial purification, the culture supernatant was first centrifuged at 6000 rpm (4 °C, 30 min), followed by filtration through a 0.22 µm membrane (Millipore, Bedford, MA, USA), and then ultrafiltration using a 30 kDa cut-off membrane (VivaFlow, Vivascience, Hannover, Germany). Protein concentration was determined using the Bradford method [38]. The partially purified *Pe*Lac was stored at 4 °C until further use.

### 2.3. Immobilization of PeLac as Protein–Inorganic Hybrids

Partially purified *Pe*Lac was immobilized using a Cu-based protein–inorganic hybrid system. Briefly, CuSO_4_ (2.0 mM) and *P*eLac (0.25 mg/mL) were mixed in 3 mL of PBS (10 mM, pH 7.2) and incubated at 4 °C for 24 h [37]. After incubation, the resulting protein–inorganic hybrid precipitates were separated by centrifugation at 10,000 rpm (4 °C, 5 min), and the residual protein concentration in the supernatant was measured. Encapsulation yield (EY), loading, and relative activity (RA) were calculated as follows (Equations (1)–(3)) [37,39]:

EY = (Amount of immobilized enzyme/Initially added enzyme amount) × 100(1)

Loading = Immobilized laccase/Weight of synthesized hybrids (2)

RA = (Activity of immobilized enzyme/Activity of free enzyme) × 100(3)

### 2.4. Assessment of Enzyme Activity

*Pe*Lac activity was evaluated spectrophotometrically using ABTS (εmax = 3.6 × 104 M^−1^ cm^−1^) as a substrate at 420 nm [37].

### 2.5. Characterization of PeLac-Based Protein–Inorganic Hybrids

The activity profiles of free and immobilized *Pe*Lac were assessed at various pH values (2.5–8.0) and temperatures (25–70 °C) using ABTS under standard assay conditions. Kinetic parameters (*K*_m_ and *V*_max_) were determined using the Michaelis–Menten model via non-linear regression analysis (Prism 5, GraphPad Software, La Jolla, CA, USA) using ABTS concentrations ranging from 0.01 to 2.0 mM at 25 °C [37]. Further, the activity of Cu–*Pe*Lac NFs was evaluated in the presence of various ions (5.0 mM), including NaCl, Ca(OH)_2_, KCl, CuSO_4_, and Na_2_CO_3_, and solvents such as ethanol and methanol (25%, *v*/*v*) under standard assay conditions.

### 2.6. Stability and Reusability

The storage stability of free and immobilized *Pe*Lac was evaluated by incubating samples at 4 °C for 30 days in their optimum pH conditions. Further, the pH stability of *Pe*Lac was evaluated at various pH levels (2.5–4.5) for a 5-day incubation period. Thermal stability was assessed by incubating the enzymes at temperatures ranging from 40 °C to 70 °C. The initial residual activity of both free and immobilized *Pe*Lac was considered 100%. Additionally, the reusability of Cu–*Pe*Lac NFs was evaluated over ten cycles under standard assay conditions [39]. Similarly, the enzyme leaching during recycling was evaluated by protein concentration in the supernatant. The leaching (%) was calculated by the amount of the protein ratios in the supernatant to the immobilized × 100 [39].

### 2.7. Measurement of Acute Toxicity

The acute toxicity of *Pe*Lac-based protein–inorganic hybrids (2.0 mg/mL) and pure Cu-NPs (1.0 mg/mL) was assessed against *V. fischeri* using an 81.9% basic toxicity test protocol by Microtox analyzer (Model 500, Modern Water, USA). Each sample was prepared with various dilutions, and its osmolarity was adjusted with an osmolarity-adjusting solution given by the manufacturer. The endpoint analysis by the Microtox assay detects the decline in the intensity of light emitted by *V. fischeri* after 30 min of exposure. The effective concentration (EC) value was represented by EC_50_, indicating a 50% reduction in *V. fischeri* luminescence after 30 min of incubation [38].

### 2.8. Degradation of Bisphenol A

The degradation of bisphenol A (50 μM) was carried out using either free (22 µg/mL) or immobilized *Pe*Lac (6.4 µg of protein/mL or 39.3 µg of NFs/mL) in 10 mL of buffer (50 mM) at various pH values (4.0–8.0) at 30 °C for 2 h under shaking conditions (100 rpm) [7]. After incubation, the reaction was terminated by adding 100 µL of HCl (0.5 M) to stop the reaction, and the resulting reaction was immediately centrifuged at 10 rpm for 5 min at 4 °C [40]. The residual bisphenol A in supernatant was analyzed via the 4-AAP coupled reaction in the presence of potassium ferricyanide under alkaline conditions that result in the formation of quinone-type dye using absorbance measurements at 506 nm, spectrophotometrically [7]. The effect of bisphenol A concentration (50–200 μM) on degradation by free and immobilized PeLac was evaluated under optimal pH for 2 h of incubation. Repeated batch degradation was conducted using 50 μM bisphenol A at 30 °C for up to ten cycles. Furthermore, degradation of 50 μM bisphenol A was investigated in the presence of potent inhibitors (0.5 mM). For real application, the degradation potential of bisphenol A (50 µM) by free and Cu–*Pe*Lac NFs was evaluated in 50% (*v*/*v*) supplementation of tap water and wastewater from the lake of Konkuk University in the buffer solution.

### 2.9. Instrumental Analysis

Field emission scanning electron microscopy (FE-SEM) images of the hybrids were obtained using an FE-SEM system (JSM-6700F, JEOL, Tokyo, Japan) [37]. Diffraction patterns of *P*eLac-based protein–inorganic hybrids were analyzed by X-ray diffraction (XRD) using an X’Pert PRO MPD diffractometer (Malvern Panalytical, Malvern, UK) [39]. Confocal laser scanning microscopy (CLSM) analysis of hybrids synthesized using FITC-labeled laccase was conducted using a confocal microscope (Olympus, Center Valley, PA, USA) as described previously [39]. The electrochemical properties of free and *Pe*Lac-based hybrids were evaluated in a 10 mL cell by cyclic voltammetry (CV) using an SP-150 potentiostat (BioLogic, Knoxville, TN, USA) with a three-electrode setup comprising a platinum wire (counter electrode), Ag/AgCl (saturated KCl, reference electrode), and a glassy carbon electrode (working electrode) [6]. A volume of 5 µL of free or *Pe*Lac (1 mg/mL) was fixed on the GCE electrode by adding nafion solution (5%, *v*/*v*) at a ratio of 9:1, and dried out for 1–2 h at room temperature. A prepared working electrode was used to analyze ABTS (0.5 mM) oxidation with a scan rate of 20 mV/s, and the potential between 0.0 and 1.3 V vs. Ag/AgCl [6]. Experimental data are presented as mean values ± standard deviations, and statistical significance was assessed using one-way analysis of variance (α = 0.05) in GraphPad Prism 5 (GraphPad Software, Inc., La Jolla, CA, USA).

## 3. Results and Discussion

### 3.1. PeLac Production and Partial Purification

The production profile of *Pe*Lac, with and without CuSO_4_ (0.2 mM), is shown in Figure S1. The highest laccase activity was observed after seven days of incubation, with 1.9 U/mL in the presence of CuSO_4_ compared to 0.14 U/mL under control conditions. Previously, *P. eryngii* var. *ferulae* was reported to produce approximately 3.0 U/mL of laccase activity [35]. In contrast, *P. eryngii* KS004 exhibited lower maximum activity, reaching only 0.38 U/mL in the presence of 1.0 mM Cu^2+^ [36]. After partial purification using 30 kDa ultrafiltration membranes, the specific activity of *Pe*Lac increased to 92.6 U/mg of total protein, with a yield of 41.9% and a purification fold of 2.1 (Table S1; Figure S2). A lower specific activity of 47.3 U/mg was previously reported for laccase isoenzymes from *P. eryngii* partially purified by ultrafiltration [34].

### 3.2. PeLac Immobilization Using Cu-Based Protein–Inorganic Hybrids

The EY and RA of laccase are strongly influenced by the protein concentration during the synthesis of protein–inorganic hybrids. Accordingly, *Pe*Lac immobilization was carried out using CuSO_4_ (2.0 mM) and enzyme concentrations ranging from 0.01 to 0.50 mg/mL at 4 °C (Table 1). After 24 h of incubation, the synthesized protein–inorganic hybrids denoted as Cu_3_(PO_4_)_2_–*Pe*Lac (Cu–*Pe*Lac) achieved an EY of 84.7%, a loading of 191 mg/g of hybrids, and an RA of up to 333%. A protein concentration of 0.25 mg/mL was found to be optimal, yielding the highest RA (333%) compared to the free *Pe*Lac activity (92.6 U/mg of protein). In contrast, purified *Trametes versicolor* laccase immobilized as Cu–laccase NFs showed a lower EY of 72.4% [8]. Laccases immobilized via protein–inorganic hybrids exhibited varying RA values depending on the metal used: (i) Zn-based hybrids achieved 86.4% RA [25]; (ii) Cu-based hybrids, ~80.0% RA [41]; (iii) Ni-based hybrids, 75.0% RA [21]; and (iv) Co-based hybrids, 60.9% RA [20]. These findings indicate that Cu–based NFS encapsulation of *Pe*Lac is highly effective in achieving high RA after immobilization.

**Figure 1:**
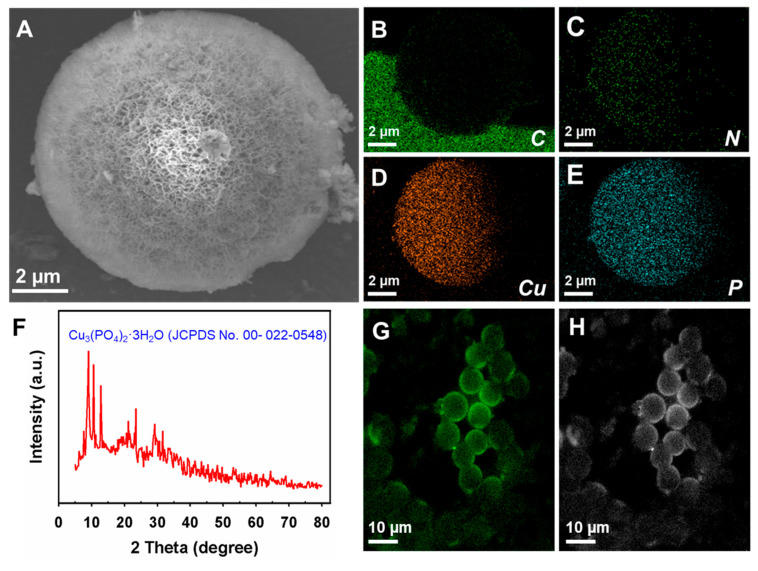
FE-SEM image of synthesized Cu–*Pe*Lac protein–inorganic hybrids (**A**); elemental mapping for carbon (**B**), nitrogen (**C**), copper (**D**), and phosphorus (**E**); XRD pattern of Cu–*Pe*Lac hybrids (**F**); and CLSM images of FITC-labeled Cu–*Pe*Lac NFs under the green fluorescence channel (**G**) and bright-field view (**H**).

FE-SEM analysis demonstrated that the synthesized Cu–*Pe*Lac protein–inorganic hybrids exhibited a flower-like morphology characteristic of NFs, with an average size of approximately 10 µm (Figure 1A). Previously reported protein–inorganic hybrids synthesized using other laccases and different metals showed varying morphologies and sizes: (i) ~50 µm for Cu–laccase hybrids with flower-like morphology [42]; (ii) ~2 µm for Co–laccase hybrids with flower-like aggregates [20]; and (iii) ~25 µm for Zn–laccase hybrids with flake-like morphology [25]. Elemental mapping confirmed the formation of Cu–*Pe*Lac hybrids by detecting the presence of Cu, carbon (C), nitrogen (N), and phosphorus (P) (Figure 1B–E). XRD analysis validated the synthesis of the hybrids by showing characteristic peaks consistent with the Cu_3_(PO_4_)_2_·3H_2_O pattern (JCPDS No. 022-0548) (Figure 1F) [37]. To further confirm *Pe*Lac immobilization, hybrids were synthesized using FITC-labeled *Pe*Lac and analyzed by CLSM measurements (Figure 1G,H). Green fluorescence emission in CLSM confirmed the presence of *Pe*Lac within NFs.

### 3.3. Characterization of Immobilized PeLac

**Figure 2:**
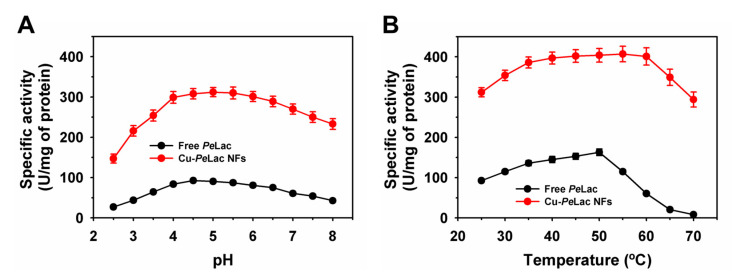
Activity profiles of free and immobilized *Pe*Lac in the form of protein–inorganic hybrids NFs at various pH values (**A**) and temperatures (**B**).

The pH activity profiles of free and immobilized *Pe*Lac are shown in Figure 2. The optimum pH values for free and Cu–*Pe*Lac NFs were 4.5 and 5.0, with a specific activity of 92.6 and 308 U/mg of protein, respectively (Figure 2A). After immobilization, *Pe*Lac exhibited a broader pH activity range, spanning from pH 2.5 to 8.0. Previous studies have reported that laccases immobilized using Co- and Cu-based NFs displayed pH optima similar to their free enzyme counterparts [20,42]. Cu–*Pe*Lac NFs demonstrated a 1.6-fold increase in RA at pH 8.0. The optimum temperatures for free and Cu–*Pe*Lac NFs were 50 °C and 55 °C with a specific activity of 163 and 404 U/mg of protein, respectively (Figure 2B). Similarly, Cu- and Co-based protein–inorganic hybrids synthesized using laccases have shown temperature optima comparable to those of the free enzyme [42,43]. Laccase immobilized via metal–organic frameworks also exhibited a similar temperature optimum to that of the free enzyme [27]. Furthermore, increasing the incubation temperature up to 70 °C led to a continuous decline in residual activity for free *Pe*Lac, whereas Cu–*Pe*Lac NFs retained significantly higher residual activity under identical conditions. At 70 °C, Cu–*Pe*Lac NFs showed a 14.2-fold higher residual activity than free *Pe*Lac. Overall, immobilized *Pe*Lac exhibited broader activity profiles across both pH and temperature conditions. These findings suggest that the properties of laccases from different sources can vary substantially following immobilization as Cu-NFs or other hybrid systems. Moreover, the use of crude rather than purified laccases may offer economic advantages for large-scale applications [20,42,43].

Crude *Pe*Lac exhibited a Michaelis–Menten constant *(K*_m_) of 118 µM and a maximum velocity (*V*_max_) of 93.1 µmol/min/mg protein toward ABTS as a substrate (Table 2). Following immobilization, the *K*_m_ value decreased significantly to 48.8 µM, indicating an increased substrate affinity. Previously, laccase immobilized via Co-based hybrids (laccase@Co_3_(PO_4_)_2_·H NFs) demonstrated a similar affinity to that of free laccase, with a *K*_m_ of approximately 3.0 µM [21]. In contrast, Zn-based laccase NFs exhibited a ~1.5-fold reduction in affinity relative to free laccase, with a Km of 6.3 µM. The *V*_max_ of *Pe*Lac increased by 3.3-fold after immobilization as Cu–*Pe*Lac NFs. Comparable *K*_m_ and *V*_max_ values were previously reported for both free and immobilized laccase using Co-based NFs and ABTS as the substrate [25]. In contrast, Cu–laccase NFs showed a ~2.0-fold reduction in substrate affinity after immobilization, with the Km increasing from 40.3 µM to 82.6 µM [42]. Additionally, *Bacillus amyloliquefaciens*-derived laccase immobilized as Cu–laccase NFs exhibited 11.1% lower catalytic efficiency than its free counterpart [41]. Electrochemical measurements revealed oxidation current peaks of 0.47 µA and 1.42 µA for free and immobilized *Pe*Lac, respectively, during the oxidation of ABTS to ABTS^+•^ at a potential of 0.5 V (Table 2). These results confirm that the enhanced catalytic performance of Cu–*Pe*Lac NFs is supported by a ~3.0-fold increase in oxidation current relative to the free enzyme. Additionally, the Cu–*Pe*Lac NFs retained residual activity of 91.1–116% in the presence of different common ions (Na^+^, Ca^2+^, Cl^−^, Cu^2+^, and CO_3_^2−^) and solvents (ethanol and methanol) (Table S2).

### 3.4. Toxicity Measurements of NFs

NPs are widely recognized as toxic materials capable of contributing to diverse forms of environmental pollution [44]. The synthesized Cu-NFs exhibited an EC_50_ value of 876 µg/mL (Table 3). In contrast, pure Cu-NPs demonstrated substantially higher toxicity, with an EC_50_ of 95.4 µg/mL. This marked difference in acute toxicity may be attributed to the larger size and distinct structural characteristics of Cu-NFs compared to Cu-NPs, which are typically ~50 nm in diameter. These findings suggest that Cu-based NFs are highly biocompatible, requiring a 9.2-fold higher concentration to achieve a 50% reduction in of *V. fischeri* viability after 30 min of incubation. Previously, nano CuO exhibited an EC_50_ of 79 µg/mL against *V. fischeri*, indicating higher acute toxicity [45]. In another study, CuO NPs showed an EC_50_ value of 258 µg/mL under similar 30 min incubation conditions with *V. fischeri* [46].

### 3.5. Stability and Reusability

**Figure 3:**
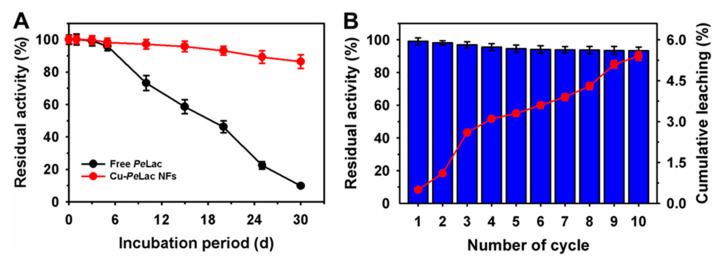
(**A**) Storage stability of free and immobilized *Pe*Lac at 4 °C for 30 days. (**B**) Reusability of Cu–*Pe*Lac NFs and cumulative leaching over ten cycles.

The thermal stability of free and Cu–*Pe*Lac NFs was initially evaluated by incubating each enzyme in its optimal pH buffer at temperatures ranging from 40 °C to 70 °C (Table 4). Under these conditions, the half-life (t_1/2_) of free *Pe*Lac ranged from 0.17 to 13.2 h, whereas Cu–*Pe*Lac NFs demonstrated an extended *t*_1/2_ of 1.12 to 62.6 h. This represents up to an 11.8-fold improvement in *Pe*Lac stability following immobilization. In contrast, Co-based *T. versicolor* laccase immobilized as aggregate protein–inorganic hybrids exhibited lower residual activity than the free enzyme after incubation at 45 °C [20]. The storage stability of free and immobilized *Pe*Lac was also assessed by incubation at 4 °C for 30 days (Figure 3A). During storage, the residual activity of free *Pe*Lac progressively declined with increasing storage duration. After 30 days, free *Pe*Lac retained only 9.9% of its initial activity. In comparison, Cu–*Pe*Lac NFs maintained a significantly higher residual activity of 86.4% under identical conditions. In contrast, Co–laccase protein–inorganic hybrids showed substantially lower stability, only a 1.4-fold improvement after 40 days of incubation compared to the control [20]. Further, on incubation at various pH levels of 2.5–4.5 during storage at 4 °C, the residual activity of Cu–*Pe*Lac decreased to 92.3% at pH 2.5 compared to 98.2% at the optimum pH of 4.5 (Figure S3). These findings suggest that the morphological structure of synthesized protein–inorganic hybrids can influence enzyme properties post-immobilization, particularly structure–stability relationships that minimize enzyme leaching and inactivation during recycling [20,42].

Under recycling assay conditions, Cu–*Pe*Lac NFs retained 94.6% and 93.3% of their initial activity after five and ten reuse cycles, respectively (Figure 3B). Under similar conditions, the cumulative leaching of 5.4% of immobilized enzyme was observed, which validates the decline in residual activity. In comparison, several immobilized hybrid systems using expensive purified laccases have demonstrated significantly lower reusability under similar conditions, with only ~30% residual activity reported for Cu–laccase NFs after ten cycles [43], ~25–40% for laccases immobilized on Ni/Zn- and Ni/Zn/Co-ferrite particles after five cycles [11], ~40% for Zn–laccase NFs after 12 cycles [42], and ~50% for laccases immobilized using magnetic dendrimer-grafted silica-coated hercynite–copper, Cu–laccase, and Co–laccase NFs after ten cycles [18,20,43].

### 3.6. Bisphenol A Degradation by Free and Immobilized PeLac

**Figure 4:**
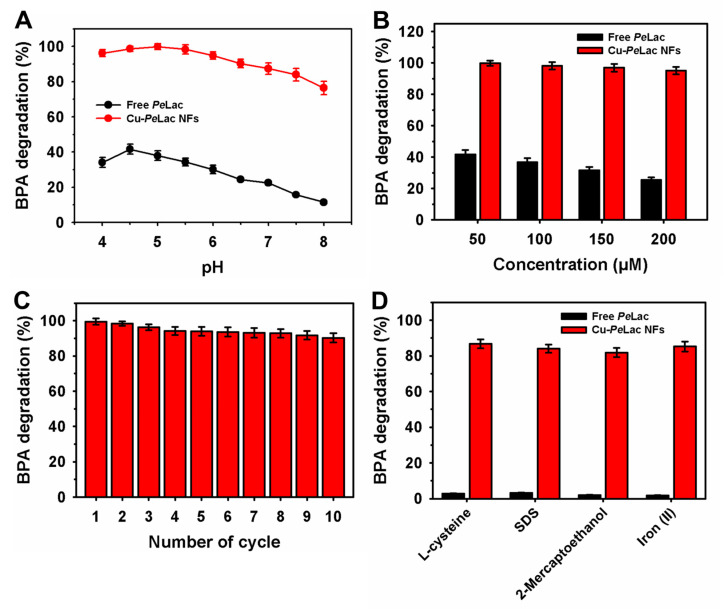
Degradation of bisphenol A by free and immobilized *Pe*Lac: (**A**) degradation at various pH levels; (**B**) degradation at different bisphenol A concentrations; (**C**) repeated-batch degradation over multiple reuse cycles; and (**D**) degradation in the presence of known laccase inhibitors.

Bisphenol A concentrations in wastewater effluents have been reported to reach up to 370 µg/L (1.6 µM), though these levels can vary significantly among different effluent sources [47]. To evaluate the potential application of laccase in xenobiotic removal, both free and immobilized *Pe*Lac were used to degrade bisphenol A. The degradation efficiency of bisphenol A and similar xenobiotics varies considerably depending on the laccase source and environmental conditions, including pH and temperature [10,48,49]. Accordingly, bisphenol A at a concentration of 50 µM was subjected to enzymatic degradation at pH values ranging from 4.5 to 8.0 for 2 h at 30 °C (Figure 4A). Free and immobilized *Pe*Lac demonstrated differing efficiencies across the tested pH range. The maximum degradation was 41.6% for free *Pe*Lac at pH 4.5 and 99.8% for Cu–*Pe*Lac NFs at pH 5.0. Compared to the free form, Cu–*Pe*Lac NFs exhibited significantly enhanced degradation capacities—2.8-fold higher at pH 4.0 and 6.6-fold higher at pH 8.0 under identical conditions. These improvements may be attributed to enhanced structural stability and the formation of a favorable microenvironment for catalysis following immobilization [19,30]. Similarly, purified *T. versicolor* laccase immobilized in Cu–alginate beads achieved 96.1% degradation of 44 µM bisphenol A at pH 5.0 [10]. In contrast, free and immobilized *T. versicolor* laccase on chitosan-functionalized halloysite nanotubes showed comparable degradation efficiencies of up to 85.0% across a broader pH range (3.0–9.0) after 12 h of incubation [30]. Additionally, laccase immobilized on a cellulose/waste Cu^2+^–activated carbon composite exhibited less than 80% degradation of bisphenol A over a pH of 3.0–7.0 [19].

When the bisphenol A concentration was increased from 50 to 200 µM, a progressive decline in degradation efficiency was observed for free *Pe*Lac, decreasing from 41.6% to 25.4% (Figure 4B). In contrast, Cu–*Pe*Lac NFs maintained high degradation efficiency, ranging from 95.1% to 99.8%, across the same concentration range. Under repeated-batch degradation conditions, Cu–*Pe*Lac NFs retained 94.0% and 90.2% degradation efficiency after five and ten reuse cycles, respectively (Figure 4C). Previously, purified laccase immobilized onto the defective metal–organic framework PCN-224 showed markedly lower degradation performance—only up to ~10% for bisphenol A concentrations ranging from 2.0 to 88.0 µM [31]. Free laccase exhibited ~2.0-fold higher degradation efficiency than its immobilized counterpart on PCN-224 at similar concentrations. Likewise, *T. versicolor* laccase immobilized in magnetic gelatin methacryloyl–chitosan hydrogel retained 73.7% degradation efficiency of 44.0 µM bisphenol A after ten reuse cycle*s* [29]. Cu–laccase NFs achieved ~80% degradation of mercaptobenzothiazole after five reuse cycles [42], while *B. amyloliquefaciens*-derived laccase immobilized as Cu–laccase NFs retained ~60% efficiency for tetracycline degradation after five reuse cycles [41]. Additionally, immobilized laccase on PCN-224 retained ~70% residual degradation of 44.0 µM bisphenol A after five cycles—closely matching the ~70% adsorption efficiency of the PCN-224 material alone [31]. Free enzyme activity is significantly inhibited by substances that limit its utility in various biotechnological applications [50,51]. Therefore, bisphenol A degradation by free and immobilized *Pe*Lac was further evaluated in the presence of known laccase inhibitors, including L-cysteine, sodium dodecyl sulfate (SDS), 2-mercaptoethanol, and Fe^2+^ ions (Figure 4D). The degradation efficiency of free *Pe*Lac declined sharply from 41.6% (without inhibitor) to 3.2%, 2.8%, 2.1%, and 1.8% in the presence of SDS, L-cysteine, 2-mercaptoethanol, and Fe^2+^, respectively. In contrast, Cu–*Pe*Lac NFs retained high bisphenol A degradation efficiencies ranging from 81.9% to 86.7% in the presence of these inhibitors. Notably, Cu–*Pe*Lac NFs demonstrated up to a 47.3-fold improvement in inhibitor tolerance compared to free *Pe*Lac. In the mechanism, the higher stability of Cu–*Pe*Lac NFs against these inhibitors might be associated with preservation of structural properties of *Pe*Lac after encapsulation or alteration in the microenvironment around the active site [39,50]. Previously, laccase immobilized on a polyacrylonitrile/polyethersulfone support exhibited lower estrogen degradation efficiency (up to 70%) in the presence of various surfactants and metal-based inhibitors [50]. In another study, bisphenol A degradation by Cu–alginate bead-immobilized laccase was reduced by more than 33% when buffer was replaced with real water [10]. Similarly, reduced bisphenol A degradation efficiency was also observed for *T. versicolor* laccase immobilized in magnetic gelatin methacryloyl–chitosan hydrogel [29]. In the real water samples analysis, a decrease of 8.2% and 12.3% in bisphenol A degradation was noted for free *Pe*Lac in tap water and wastewater, respectively, as compared to the control (Figure S4). In contrast, a lower than 1.0% decline in bisphenol A degradation was recorded for Cu–*Pe*Lac NFs under similar conditions.

## 4. Conclusions

The selection of a suitable support material plays a critical role in determining the overall effectiveness of enzyme immobilization, as it significantly influences the structural and catalytic properties of the resulting biocatalyst for diverse biotechnological applications. This study reports, for the first time, the efficient immobilization of *Pe*Lac using copper-based protein–inorganic Cu–*Pe*Lac NFs for the degradation of bisphenol A in the presence of potent enzyme inhibitors. Following immobilization, the Cu–*Pe*Lac NFs exhibited enhanced catalytic performance across a wide pH and temperature range, significantly improved stability, and higher degradation efficiency compared to free *Pe*Lac. The Cu–*Pe*Lac NFs also retained substantial activity in the presence of known inhibitors, demonstrating enhanced robustness and reusability. Furthermore, the synthesized Cu–*Pe*Lac NFs showed superior biocompatibility against *V. fischeri* relative to Cu-NPs. These findings highlight an eco-friendly strategy for immobilizing crude laccases from diverse biological sources and underscore their potential for broad applications in environmental remediation, particularly in the degradation of xenobiotics.

## Figures and Tables

**Table 1:** Immobilization of *Pe*Lac using Cu-based protein–inorganic hybrids at various protein concentrations.

Protein (mg/mL)	Encapsulation Yield (%)	Loading (mg/g of Synthesized Hybrids)	Relative Activity (%) ^a^
0.05	84.7 ± 2.5	75.6 ± 5.6	215 ± 16.6
0.10	80.2 ± 2.7	97.8 ± 6.3	278 ± 18.7
0.25	78.8 ± 2.9	163 ± 9.5	333 ± 21.9
0.50	54.5 ± 4.1	191 ± 11.6	205 ± 17.0

^a^ Relative activity was calculated based on free *Pe*Lac activity, where 92.6 U/mg of total protein was considered 100%.

**Table 2:** Kinetic parameters and electrochemical properties of free and immobilized *Pe*Lac as protein–inorganic hybrids.

Enzyme	*V*_max_ (µmol/min/mg Protein)	*K*_m_ (µM)	Oxidation Current Peak (µA) ^a^
Free *Pe*Lac	93.1 ± 6.1	118 ± 8.9	0.47 ± 0.03
Cu–*Pe*Lac NFs	311 ± 19.3	48.6 ± 3.3	1.42 ± 0.07

^a^ Measured at a potential of 0.5 V and a scan rate of 20 mV/s.

**Table 3:** Acute toxicity of Cu-NPs and Cu-based protein–inorganic hybrids NFs.

Particles	EC_50_ Concentration (µg/mL)
Cu-NPs	95.4 ± 6
Cu-NFs	876 ± 48

**Table 4:** Thermal deactivation rate constant (*k*_d_) and half-life *(t*_1/2_*)* of free and immobilized *P*eLac as protein–inorganic hybrid NFs.

Temperature (°C)	Free *Pe*Lac		Cu–*Pe*Lac NFs	
	*k*_d_ (h^−1^)	*t*_1/2_ (h)	*k*_d_ (h^−1^)	*t*_1/2_ (h)
40	0.05 ± 0.003	13.2 ± 1.03	0.01 ± 0.001	62.6 ± 3.2
45	0.10 ± 0.007	6.93 ± 0.52	0.02 ± 0.001	36.8 ± 1.9
50	0.25 ± 0.015	2.78 ± 0.19	0.04 ± 0.002	19.2 ± 1.1
55	0.66 ± 0.028	1.05 ± 0.07	0.06 ± 0.004	12.3 ± 0.8
60	1.05 ± 0.049	0.66 ± 0.03	0.09 ± 0.005	7.81 ± 0.6
65	2.23 ± 0.088	0.31 ± 0.02	0.24 ± 0.013	2.86 ± 0.3
70	4.08 ± 0.123	0.17 ± 0.01	0.62 ± 0.033	1.12 ± 0.2

## Data Availability

The original contributions presented in this study are included in the article/supplementary material. Further inquiries can be directed to the corresponding author.

## Supplementary Materials

The following supporting information can be downloaded at: https://www.mdpi.com/article/10.3390/jox15040108/s1, Figure S1: *Pleurotus eryngii* laccase production profile with and without CuSO_4_ (0.2 mM); Figure S2: SDS-PAGE analysis of *Pleurotus eryngii* crude enzymes: M—Protein marker, lane 1—Enzymes treated with 30 kD spin-column; Figure S3: Storage stability (4 °C) of immobilized *Pe*Lac at various pH levels for 5 days; Figure S4: Influence of tap and wastewater supplantation in buffer on degradation of bisphenol by free and Cu–*Pe*Lac NFs; Table S1: Partial purification of *Pleurotus eryngii* laccase; Table S2: The influence of common ions and organic solvents on the activity of Cu-*Pe*Lac NFs.
