# Supplementary material for: Immobilization of Pleurotus eryngii Laccase via a Protein–Inorganic Hybrid for Efficient Degradation of Bisphenol A as a Potent Xenobiotic
Source: J Xenobiot. 2025 Jul 3;15(4):108. doi: 10.3390/jox15040108 (PMC12286210; doi:10.3390/jox15040108)
Supplement: Supplementary file 1 [file jox-15-00108-s001.zip › jox-3677985-supplementary.pdf]

Immobilization of *Pleurotus eryngii* Laccase via a Protein–Inorganic Hybrid for Efficient Degradation of Bisphenol A as a Potent Xenobiotic

Sanjay K. S. Patel [1,2], Rahul K. Gupta [1,] and Jung-Kul Lee [1,*]

[1] Department of Chemical Engineering, Konkuk University, Seoul 05029, Republic of Korea

[2] Department of Biotechnology, Hemvati Nandan Bahuguna Garhwal University (A Central University), Srinagar 246174, Uttarakhand, India

* Correspondence: krhee@konkuk.ac.kr (J.K.L.)

Table S1. Partial purification of *Pleurotus eryngii* laccase.

Purification step	Volume (mL)	Total protein (mg)	Specific activity (U/mg total protein)	Yield (%)	Fold purification
Crude	100	4.3	44.2	100	1
30kD purification	3.7	1.8	92.6	41.9	2.1

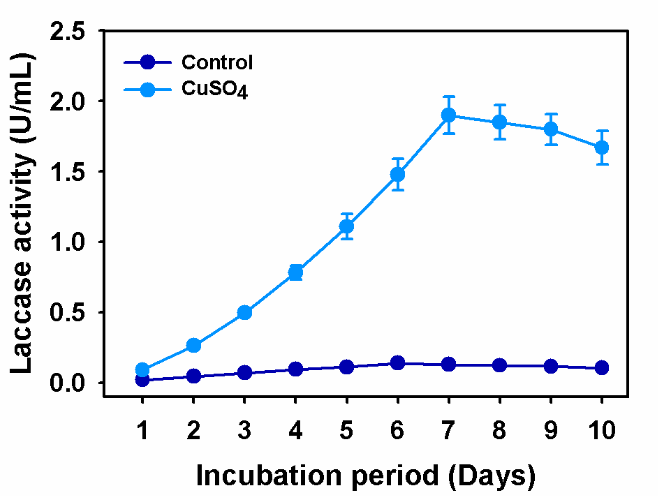

Figure S1. *Pleurotus eryngii* laccase production profile with and without $CuSO_4$ (0.2 mM).

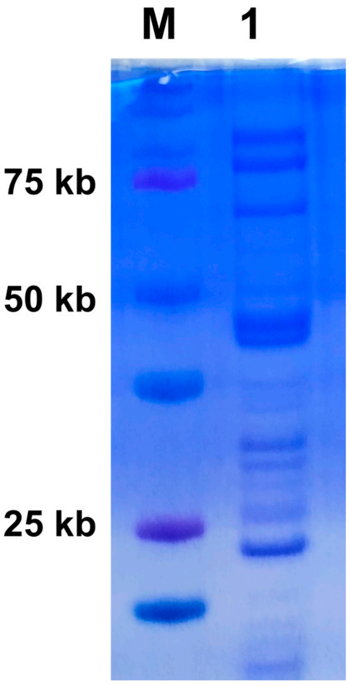

Figure S2. SDS-PAGE analysis of *Pleurotus eryngii* crude enzymes: M - Protein marker, lane 1 – Enzymes treated with 30 kD spin-column.

editage

Editing Certificate

This document certifies that the manuscript listed below has been edited to ensure language and grammar accuracy and is error free in these aspects. The edit was performed by professional editors at Editage, a brand of Cactus Communications. The author's core research ideas were not altered in any way during the editing process. The quality of the edit has been guaranteed, with the assumption that our suggested changes have been accepted and the text has not been further altered without the knowledge of our editors.

MANUSCRIPT TITLE

Immobilization of Pleurotus eryngii Laccase via a Protein–Inorganic Hybrid for Efficient Degradation of Bisphenol A as a Potent Xenobiotic

AUTHORS

Jungkul Lee

ISSUED ON

May 16, 2025

JOB CODE

KOUNI_6025

THE EDITAGE Promise QUALITY GUARANTEE

Prabh Grewal
Senior Vice President - Editage

editage helping you get published

Since 2002, Editage has helped over 430,000 authors publish around 1.2 million research papers in scholarly journals across over 1000 disciplines through editorial, translation, transcription, and publication support services. Editage is a brand of Cactus Communications (cactusglobal.com), a science communication and technology company.

CACTUS

GLOBAL : +1(669) 272-1214 | request@editage.com

KOREA : 02-3478-4396 | submit-korea@editage.com

editage.com | editage.co.kr | editage.jp | editage.cn | editage.com.br | editage.com.tw | editage.de
